# Neuroanatomical Correlates of Cognitive Dysfunction in 22q11.2 Deletion Syndrome

**DOI:** 10.3390/genes15040440

**Published:** 2024-03-30

**Authors:** Simon Smerconish, James Eric Schmitt

**Affiliations:** Departments of Radiology and Psychiatry, Division of Neuroradiology, Hospital of the University of Pennsylvania, Philadelphia, PA 19104, USA

**Keywords:** 22q11.2 deletion syndrome, cognitive phenotype, neuroimaging, MRI

## Abstract

22q11.2 Deletion Syndrome (22q11.2DS), the most common chromosomal microdeletion, presents as a heterogeneous phenotype characterized by an array of anatomical, behavioral, and cognitive abnormalities. Individuals with 22q11.2DS exhibit extensive cognitive deficits, both in overall intellectual capacity and focal challenges in executive functioning, attentional control, perceptual abilities, motor skills, verbal processing, as well as socioemotional operations. Heterogeneity is an intrinsic factor of the deletion’s clinical manifestation in these cognitive domains. Structural imaging has identified significant changes in volume, thickness, and surface area. These alterations are closely linked and display region-specific variations with an overall increase in abnormalities following a rostral-caudal gradient. Despite the extensive literature developing around the neurocognitive and neuroanatomical profiles associated with 22q11.2DS, comparatively little research has addressed specific structure–function relationships between aberrant morphological features and deficient cognitive processes. The current review attempts to categorize these limited findings alongside comparisons to populations with phenotypic and structural similarities in order to answer to what degree structural findings can explain the characteristic neurocognitive deficits seen in individuals with 22q11.2DS. In integrating findings from structural neuroimaging and cognitive assessments, this review seeks to characterize structural changes associated with the broad neurocognitive challenges faced by individuals with 22q11.2DS.

## 1. Introduction

The 22q11.2 Deletion Syndrome (22q11.2DS), arises from a hemizygous deletion on the long arm of chromosome 22q11.2 [1]. With a prevalence of approximately 1 in 3000–6000 live births, 22q11.2DS comprises the most common chromosomal microdeletion in humans and presents with a varied combination of anatomical, behavioral, and cognitive abnormalities [2,3]. Anatomical problems most commonly observed in 22q11.2DS include cardiac defects, palatal deformities, and facial abnormalities [4]. The extant literature has also demonstrated that individuals with 22q11.2DS stand at increased risk for an array of psychiatric disorders, most notably schizophrenia, though Autism Spectrum disorder (ASD), Attention Deficit Hyperactivity Disorder (ADHD), and Anxiety disorders are among the most prevalent [5]. The cognitive impacts and psychiatric comorbidities (e.g., ADHD) associated with 22q11.2DS have been extensively studied. Similarly, structural brain imaging has been used for decades for both research and clinical care of this condition. Though not explicitly recommended in the clinical care guidelines for patients without seizures or specific neurological signs and symptoms, many individuals with the 22q deletion have brain imaging at some point in their treatment [6]. However, though research using structural magnetic resonance imaging (sMRI) has increasingly characterized the 22q11.2 neuroanatomic endophenotype, comparatively less research has addressed distinct structure–function relationships explaining the unique cognitive phenotype of individuals with 22q11.2DS.

This paper aims to offer a review of both the cognitive and neuroanatomical profiles associated with 22q11.2 deletion syndrome, as well as the state of research attempting to identify structure–function relationships. We begin with a summary and analysis of both broad deficits and specific challenges in cognitive functioning associated with the 22q11.2 deletion, weighing the extent of impact across affected domains and noting topics requiring additional analysis. Following an overview of the many manifestations of cognitive dysfunction in 22q11.2DS, we proceed to review the characteristic morphological differences, primarily in cortical volumes, thickness, and surface area, distributed throughout the brain in both cortical and subcortical regions as well as the cerebellum. We close with an assessment of the nascent literature associating specific morphological abnormalities with aberrant cognitive functioning including findings from populations sharing aspects of both the phenotypic and anatomical profiles. In integrating these two largely disparate bodies of literature and categorizing the limited research attempting to bridge this gap, the review fundamentally attempts to assess the degree to which structural findings can explain cognitive deficits in 22q11.2DS, and highlights areas necessitating further inquiry.

## 2. Cognitive Profile in 22q11.2DS

### 2.1. Overall Intellectual Functioning

While there is considerable heterogeneity in the distribution of cognitive dysfunction in individuals with 22q11.2DS across specific cognitive domains, intelligence quotient (IQ) deficits are among the most common features of the shared cognitive profile, with most children showing borderline to moderate cognitive disability [7,8,9]. Studies in smaller cohorts initially found that there was a significant 6–8 point increase in verbal IQ (VIQ) over performance IQ (PIQ), consistent with trends found in non-verbal learning disability [7,8,9,10]. However, more recently in a large sample of 1478 subjects, Zhao et al. [11] identified a statistically significant but more modest 3-point increase in favor of VIQ. A meta-analysis by Moberg et al. [12] replicates this finding but additionally notes that a significant PIQ-VIQ difference is only observed in pediatric samples, though verbal domains are comparatively less impacted than performative across a variety of skills.

The extant literature on the influences of sex and age on cognitive functioning in 22q11.2DS is somewhat inconsistent. Minor sex differences in full-scale IQ (FSIQ) have been reported in 22q11.2DS, with several studies reporting FSIQ in females is higher than in males [13,14]. For example, Zhao et al. [11] found that females have a significantly 1–2 point higher FSIQ than male subjects in their sizable cohort. In contrast, other groups have found no significant effect of sex [9]. The effects of age are difficult to assess since longitudinal studies are limited in 22q11.2DS, though a few studies have found an atypical pattern of premature cognitive decline [14,15,16]. Other groups have found no evidence of age-related cognitive decline [17,18]. It has been argued that apparent age-related effects of 22q11.2DS may be either attributable to a constrained age window in prior studies or owed to the expected declines in FSIQ coinciding with the emergence of psychotic features and other psychiatric illnesses in early adolescence. Research has also categorized the relationship between deletion size and FSIQ in individuals with 22q11.2DS, with findings by Zhao et al. [11] demonstrating a significant deficit of 6.25 points associated with the longer (AD) deletion relative to the shorter (AB) deletion. In summary, the current literature on global cognitive impairments in 22q11.2DS suggests small age effects, the influence of developmental and psychiatric factors influencing the VIQ-PIQ gap, conflicting results on neurodevelopmental associations with global functions (but some evidence of relatively stable deficits in FSIQ across development after accounting for psychiatric conditions), and significant impacts of more recently identified genetic moderators like deletion size.

### 2.2. Executive Functioning and Attentional Processes

The fundamental organization of executive functions remains a topic of ongoing debate [19,20], though assessing 22q11.2DS subjects using the widely accepted hierarchical model proposed by Miyake et al. [21] reveals marked deficits spanning all of the principal domains: working memory (Updating), cognitive flexibility (Shifting), and inhibitory control (Inhibition). Individuals with 22q11.2DS encounter related challenges in higher-order executive functions, such as planning and multitasking, primarily due to deficits in these core executive domains—working memory, cognitive flexibility, and inhibitory control—which are further impacted by distinct attentional impairments [22]. Deficient executive capacities contribute to the distinctive neuropsychological profile in 22q11.2DS and are partially distinct from general intellectual functioning and exhibit unique developmental trajectories [18,23].

All core domains identified in the hierarchical model of executive functioning are demonstrably impacted in individuals with 22q11.2DS. Deficits in working memory are consistently reported across different testing modalities [24,25,26,27]. Similarly, cognitive flexibility is notably compromised, with 22q11.2DS subjects exhibiting difficulties in adapting to changes in task paradigms [9,24,28]. Inhibitory control is another domain where individuals with 22q11.2DS show significant challenges, struggling with both response suppression and the appropriate initiation of responses [29,30]. These findings collectively suggest that all core executive functions are adversely impacted by the deletion.

Deficits in executive functioning are exacerbated by deficits in attention. Individuals with 22q11.2DS exhibit general attentional problems, including deficits in sustained and selective attention, as well as difficulties in task shifting [24,26,31]. Spatial attention has been found to be a particularly impacted area, with 22q11.2DS subjects experiencing difficulty in dynamically shifting their attention in the presence of distractors [9,31,32]. Documented impairments in planning and multitasking abilities, both considered higher-order executive functions, suggest deficits in both central executive functions and attentional control in 22q11.2DS. Deficits in planning in individuals with 22q11.2DS were found to be significantly associated with working memory deficits [24,25]. Significant challenges in sustaining concentration and attention on task execution were also observed. Diminished attention was observed to be proportional to deficits in overall intellectual function. Schneider et al. [33] identified impaired multitasking abilities that were attributable to a combination of deficits in working memory and difficulties maintaining concentration on task execution rather than overall intellectual functioning. This additional layer of difficulty in higher-order executive functions suggests a more complex cognitive deficit in 22q11.2DS, impacting not just isolated cognitive processes but also the integration and pragmatic application of these processes in daily life.

### 2.3. Perceptual and Motor Skills

The perceptual and motor skills domain in 22q11.2DS is characterized by notable visuospatial difficulties. Studies collectively indicate that individuals with this syndrome face significant challenges in spatial processing and object localization, affecting their ability to monitor and adaptively interact with their environment [9,26,34,35]. Spatial attention, a key component of visuospatial functioning, is particularly compromised; 22q11.2DS subjects struggle with maintaining and shifting visual attention, especially in the presence of distractors, highlighting a fundamental issue in executive functioning related to spatial tasks [31,32,35].

Additionally, individuals with 22q11.2DS exhibit significant visuomotor deficits in both psychomotor speed and fine motor control and precision, compared to typically developing peers, and independent of concurrent IQ deficits [35,36]. Impaired visuospatial memory may contribute to difficulties integrating visual information with appropriate motor responses, with evidence that individuals with 22q11.2DS experience substantial difficulties in retaining and manipulating visual information [9,24,34,37].

Additionally, mathematical skills are adversely affected, with individuals with 22q11.2DS showing impairments in arithmetical computation, counting, and other aspects of numeracy [35]. These challenges in mathematical abilities are believed to stem from, or be exacerbated by, underlying deficits in spatial memory and attentional control and contribute to diminished levels of academic achievement among individuals with the deletion [8,9,34,35]. The influence of these deficits is particularly evident in tasks requiring timed performance, underscoring the need for tailored educational and cognitive support strategies that account for the additional time required for task execution [35].

### 2.4. Language Processing Abilities

The spectrum of language processing deficits in 22q11.2DS includes overarching difficulties in verbal reasoning, memory, learning, and attention, as well as more focal issues in phonology, syntax, and semantics, all critically impacting vocabulary development and speech production. Verbal intelligence and reasoning are areas of notable concern, with substantial challenges in these domains evidenced by deficits in abstract verbal reasoning and list memory tasks [26]. These difficulties in verbal cognitive operations are also associated with compromised verbal attention [9].

Verbal memory in 22q11.2DS is differentially characterized in the literature, with some studies suggesting it as a relative strength and others documenting pronounced deficits. While Woodin et al. [9] and Lajiness-O’Neill et al. [38] indicate that verbal memory is significantly less impacted than visuospatial memory, research by Maeder et al. [29] and Latrèche et al. [39] depicts a less optimistic picture, finding significant challenges in both immediate and delayed verbal memory. In combination, these deficits in both immediate and delayed verbal memory suggest a pattern of forgetting that may point to an impaired process of memory consolidation [29]. Findings suggest that there is variability, likely attributable to age, with some individuals with 22q11.2DS displaying verbal memory capabilities within normal ranges [34].

Deficits may extend to verbal learning, though divergent findings suggest both its preservation and significant decline within individuals with 22q11.2DS [29,39]. Furthermore, the broader spectrum of language skills, which includes phonology, syntax, and semantics, is also affected [40]. Deficits in these areas come as individuals with 22q11.2DS often exhibit clinical deficits across language domains indicative of developmental lag, evident in delayed vocabulary acquisition and speech production abilities [8,40]. This varied presentation within the domain of language processing capabilities merits further study across development, especially given the skills’ fundamental contribution to academic achievement and social development.

### 2.5. Socioemotional and Behavioral Functioning

Socio-cognitive difficulties in 22q11.2DS span challenges in skills like facial recognition, emotional attribution, and theory of mind-related processes, and contribute to deficits in measures of real-world social functioning and peer interaction. Memory for faces, a key aspect underlying social interaction, is notably deficient, as individuals with 22q11.2DS demonstrate significant difficulty with tasks designed to assess facial recognition and image retention [26]. Additionally, individuals with the deletion are significantly less accurate in facial-emotional recognition, exhibiting deficits beyond those typically seen in schizophrenia [41,42].

Individuals with 22q11.2DS experience significant challenges in processes relating to theory of mind (ToM), and the ability to recognize and reason about the mental states of others [25]. Difficulty with ToM is demonstrated by diminished performance on mentalization and emotional attribution tasks, fundamental skills for navigating social situations [25,43]. Unlike specific difficulties in facial and emotional recognition, many studies have acknowledged ToM deficits to be partially attributable to global cognitive deficits, deficient executive functioning, or attentional problems rather than constituting an independent and acutely impaired ability [25,42].

Socio-cognitive difficulties experienced by individuals with 22q11.2DS extend to emotional recognition and attribution, where there is a significant disconnect in interpreting and reacting appropriately to others’ emotional expressions, a skill closely tied to ToM deficiencies [25,42,43]. It remains unclear to what degree socio-cognitive and behavioral dysfunction and the resulting impact on measures of real-world social behavior and peer interaction are attributable to acute difficulties in cognitive processes like emotional attribution, behavioral features relating to comorbid psychiatric disorders like hyperactivity, or broad intellectual functioning deficits [25,42,43]. Thus, social difficulties in individuals with 22q11.2DS, evident in reported challenges in maintaining peer relationships and parental reports of poorer real-world social functioning, are likely the result of multiple co-occurring processes negatively impacting individuals’ social skills [25,43].

### 2.6. Developmental Trajectories in Cognition

Differing interpretations in the literature regarding IQ’s developmental pattern in 22q11.2DS exemplifies the difficulty in discerning age effects given limited longitudinal analyses and the high prevalence of psychiatric disorders, notably psychotic disorders, which have documented prodromal and concurrent impacts on IQ [44,45,46]. Some authors have found evidence of premature cognitive decline in individuals with 22q11.2DS [14,16,47], while others argue developmental lag [48] or developmental deficit [17] better describe the temporal changes in cognitive abilities. While there is a clear consensus as to the average thirty-point deficit in FSIQ seen in 22q11.2DS subjects at baseline assessment [7], the improvement in global cognitive functioning in comparison to normative developmental patterns is still a topic of considerable debate. A trend that has emerged with relative unanimity is that on average, likely driven by a subset of subjects that go on to develop a psychotic disorder or display precursor symptoms, individuals with 22q11.2DS display a decline in VIQ beginning in early adolescence which might lead to the conclusion of age-related cognitive decline [16,17,49]. Given the correspondence of this discrete period in early adolescence with the onset of psychotic symptoms in some 22q11.2DS subjects, who display sharper declines in VIQ and consequently FSIQ during this period, there is considerable difficulty in disentangling developmental patterns of IQ development attributable to the onset of a psychotic disorder versus one attributable to the overall cognitive profile of the deletion [16,17,47].

Longitudinal research on the development of executive functions and focal attention in 22q11.2DS is limited, yet findings consistently demonstrate widespread deficits in executive function and evidence of developmental delay or lag varying across specific capacities [18,30,50]. For instance, functions such as visual attention, inhibition, cognitive flexibility, updating, and initiation, while not demonstrating a pattern of developmental maturation (wherein individuals with 22q11.2DS would catch up to non-deleted subjects), do display improvements across development. However, results differ as to whether the performance gap in comparison to typically developing (TD) controls widens following a pattern of developmental lag or performance remains depreciated but with a comparable upward trajectory indicative of developmental deficit [17,18,50]. Conclusions are limited by the difficulty of retaining subjects across longitudinal studies, partly the result of severe functional declines associated with psychiatric illness, with many individuals often excluded from analyses encompassing later age windows thus introducing the potential for ascertainment biases [12]. Nevertheless, longitudinal analyses including varied neurocognitive assessment paradigms across development are essential to make results differentiable from normative developmental fluctuations [17] as well as mitigate the impact of different testing modalities [18]. The influence of testing modality is evident in Maeder et al.’s [18] sample, wherein only initiation and inhibition showed a constant developmental pattern, with other tests of focal attention, updating, and cognitive flexibility demonstrating different patterns dependent on the specific features of the respective measures used in analyses.

Largely influenced by the onset of comorbid psychiatric conditions, there is notable heterogeneity in the development of cognitive functioning between individuals sharing the 22q11.2 deletion. With respect to cognitive development, psychiatric comorbidity, particularly the cognitive impacts of psychotic disorders, creates discrete subgroups within the 22q11.2DS population, making it challenging to define a singular profile of developmental patterns of cognitive abilities [16]. Developing an understanding of the patterns of cognitive development in individuals with the deletion alone versus those with a psychiatric condition not only allows for the consideration of additional indicators of the later emergence of a given disorder but also allows for 22q11.2DS subjects to contribute to the broader literature for a given psychiatric diagnosis, assuming that the effect of the deletion on cognitive development can be accounted for. Research has revealed significant discontinuities in cognitive development in 22q11.2DS subjects compared to typical developmental trajectories, particularly across domains of executive functioning. These observations highlight the need for further longitudinal studies to identify developmental patterns respective of discrete subgroups often delineated by comorbid psychopathology, in order to maximize the utility of 22q11.2DS as a model for psychosis as well as speak to common patterns of cognitive development associated with the deletion.

### 2.7. Impact of Psychiatric Comorbidity

In individuals with 22q11.2DS, the manifestation and development of cognitive functions are intricately linked to the presence of comorbid conditions, especially schizophrenia and other psychotic disorders for which 22q11.2DS is widely used as a genetic model. The respective influences of the striking prevalence of psychotic, learning, mood, and anxiety disorders in 22q11.2DS further complicate attributions of cognitive challenges in light of analyses like Yi et al. [51], wherein approximately 50% of 22q11.2DS subjects were found to have two or more concurrent psychiatric diagnoses and that collective psychiatric burden, as well as the independent presence of psychosis spectrum disorders, contributed to lower global functioning. The high prevalence of individuals with 22q11.2DS with multiple co-occurring disorders somewhat diminishes the utility of using 22q11.2DS as a genetic model for schizophrenia and other psychotic disorders in the difficulty in parsing out each disorder’s specific neurological, cognitive, and behavioral impact. However, psychotic disorders, in particular, have been recognized in the extant literature to have widespread impacts on cognitive abilities before the emergence of overt psychosis, differentiable from the challenges associated with the deletion alone, and potentially distinct from idiopathic psychosis spectrum disorders.

An array of cognitive measures in individuals with 22q11.2DS have emerged as reliable predictors of the later emergence of psychotic disorders. Childhood deficits in executive functioning have been found to predict the subsequent emergence of positive symptoms of psychosis in adulthood [52]. Similarly, Chawner et al. [53] identified deficits in executive functioning and attention during childhood to be predictors of the emergence of Psychosis Spectrum Conditions (PSC) in early adulthood. Lower FSIQ at baseline is also linked to a higher likelihood of developing psychosis later in life [16]. More intricate developmental patterns, specifically in reading and emotional recognition abilities that weaken in childhood, improve during adolescence, and then decline in young adulthood, are also predictive of prodromal or overt psychosis [54,55].

When comparing 22q11.2DS individuals with and without psychosis, those with psychotic disorders generally exhibit significantly lower FSIQ scores, with particular deficits in Verbal IQ, implicating the challenges in abstract verbal reasoning associated with thought disorders [47]. A steeper decline in FSIQ, focally driven by decrements in VIQ, is observed in children with both 22q11.2DS and psychosis as compared to a lesser decrement in those without [16]. The presence of a psychotic spectrum disorder has been documented to have an additive effect on the underlying cognitive difficulties exhibited by 22q11.2DS subjects with replicated associations to FSIQ decrements [39,51].

Comparing the cognitive profiles of 22q11.2DS individuals with those with idiopathic psychosis or schizophrenia reveals both significant areas of convergence as well as potential differences. The overall cognitive profile of 22q11.2DS is similar to that of schizophrenia, but with notable additional challenges, such as poorer attention, visuospatial processing, emotional recognition, and verbal processing, although response speed is less affected in 22q11.2DS [26]. Lower social cognitive abilities in 22q11.2DS subjects compared to those with Clinical High Risk (CHR) or First Episode Psychosis (FEP) could be due to the declines observed in the prodromal conversion to psychosis or may be attributable to lower FSIQ in individuals with 22q11.2DS in comparison to other similarly vulnerable groups [43]. Significantly weaker emotional recognition abilities are observed in 22q11.2DS subjects compared to schizophrenia patients, suggesting potential similarities in related deficits in Theory of Mind [42]. Examining cognitive precursors, consequences, and contrasts in 22q11.2DS with psychosis reveals how psychiatric comorbidity profoundly alters the cognitive trajectory in individuals with 22q11.2 deletion syndrome, who already demonstrate atypical development of cognitive functions and show decrements across a diverse array of cognitive domains.

## 3. Structural Neuroimaging Findings in 22q11.2DS

### 3.1. Total Brain and Lobar Volumes

Global and lobar brain volume reductions in 22q11.2DS were first identified over two decades ago in small samples using region of interest (ROI) based parcellations and magnetic reasoning imaging (MRI). Global volumetric reductions in both grey and white matter have been described in numerous prior studies [35,56,57,58]. Initial studies suggested disproportionate volume loss in parietal and occipital lobes. Subsequent meta-analyses by Tan et al. [59] and Rogdaki et al. [60] largely confirmed initial volumetric findings but in much larger samples (containing 227 and 375 subjects, respectively). Findings across both of these meta-analyses largely converge, with volumetric reductions generally observed in 22q11.2DS along a rostral-caudal gradient, with the posterior cerebrum most impacted and frontal regions relatively preserved. Results in Rogdaki et al. [60], were somewhat more nuanced, finding relatively spared frontal lobes, but no statistically significant reductions in the occipital or parietal lobes. Relatively weak statistical significance for specific subregions may be partly owed to a limited number of studies performing parcellations of these areas.

More recently, studies have tended to examine neuroanatomic differences in cerebral volumes at higher spatial resolution. Jalbrzikowski et al. [61] identified significantly reduced gyral-level ROI volumes in the superior parietal cortex, inferior temporal cortex, fusiform gyrus, anterior cingulate, and right middle frontal and precentral cortex in 31 subjects with 22q11.2DS compared to a TD control population. In an analysis of 53 subjects using vertex-level analyses, Schmitt et al. [62] found similar patterns despite using an independent sample and distinct image-processing strategy, with the most significant decreases in cortical volume in the rostral lateral frontal lobe, superior parietal lobe, and near the temporal pole. Gudbrandsen et al. [63] performed vertex-level analysis in 62 subjects with 22q11.2DS and also found posterior-predominant reductions in cortical volume in occipital and parietal lobes and cingulate, but no significant reductions in lateral frontal lesions. This study also found small regions of significantly increased volumes in the insula and posterior superior frontal gyrus.

Considered in aggregate, these studies indicate that cerebral volumes are globally reduced in individuals with 22q11.2DS relative to TD controls, with less pronounced regional variation at lobar and gyral levels. Numerous studies of cortical volumes (i.e., measures excluding subcortical white matter) have been fairly consistent in identifying posterior cerebral and limbic involvement at both gyral and vertex levels of spatial resolution. In general, cortical volumes are driven by a combination of cerebral surface area (SA) and cortical thickness (CT), with the former usually dominating the measure of CV (since it accounts for two spatial dimensions). Gudbrandsen et al. [64] found that most decreases in cortical volume were driven by decreases in SA, whereas most increases in 22q11.2DS were due to increased CT relative to TD controls. Attributions as to the nature and causes of decreases in whole and cortical brain volume (CV) have evolved over time, with a growing literature assessing patterns of cortical thickness (CT) and surface area (SA) in 22q11.2DS (described in more detail below). For instance, an analysis of 474 deleted individuals by Sun et al. [65] suggests reductions in posterior surface area relative to anterior regions likely underlie findings of a rostral-caudal gradient in volumetric reductions.

It is important to note that 22q11.2DS is a complex, multi-system condition, a fact that is not commonly accounted for in neuroimaging and neuropsychiatric studies. In particular, despite a high prevalence of congenital heart defects (CHD) and other cardiovascular abnormalities in 22q11.2DS, CHD is often not controlled for. It has been theorized that decreased CNS perfusion caused by CHD is responsible for the observed changes in total cerebral, cortical, and hippocampal volumes, as well as in characteristic patterns of aberrant cortical gyrification [66,67,68]. Given the presence of CHD is seldom controlled in large analyses of cortical morphology in individuals with 22q11.2DS, it poses a major potential confound that is difficult to separate from direct neuroanatomic effects on cognitive and motor deficits in this condition [69].

### 3.2. Cortical Thickness and Surface Area

Global measures of CT are significantly increased in 22q11.2DS and SA is substantially decreased [62]. Furthermore, modern parcellation and segmentation algorithms allow for the isolation of the cortical sheet from the remainder of the cerebrum and facilitate high-resolution measurements at the cortical surface. Characteristic patterns of CT in 22q11.2DS have been observed, with recent analyses helping to clarify its relationship to both cortical SA and CV [64,65,70]. Individuals with 22q11.2DS typically exhibit a pattern of overall cortical thickening, widely distributed across diffuse areas of the brain bilaterally [61,62,64,65]. It is notable that a higher CT relative to TD is an unusual finding in psychiatric and neurogenetic disorders, but this observation has been consistently replicated in 22q11.2DS and may be indicative of neuromigrational anomalies. While CT is generally greater in 22q11.2DS over most of the cerebrum, specific regions of significantly thinner cortex compared to TD controls have also been independently observed, specifically in the superior temporal gyrus (STG) and cingulate [62,65]. These regions are of particular interest since both have associations with schizophrenia [71,72]. In the largest study to date on 474 subjects in the ENIGMA-22 consortium, Sun et al. [65] found the superior temporal, cingulate, and parahippocampal cortex CT were all significantly decreased in 22q11.2DS, despite more widespread relative increases in CT elsewhere in the cerebrum.

There is some evidence that CT and SA are genetically orthogonal in adult TD populations [70,73], although there is also evidence of greater genetic overlap in TD children and associations are likely dynamic (Schmitt et al. [74]). Reductions in SA in 22q11.2DS largely mirror the observed increases in CT and likely share an overlapping root cause [61,62,64,65]. Sun et al. [65], found reductions in SA had similar patterns to CT, with the former having roughly double the effect size. The authors suggest that a rostral-caudal gradient in volumetric reductions were primarily owed to increased reductions in SA along the same axis.

### 3.3. Specific Regional Abnormalities

#### 3.3.1. Frontal Lobe

While initial MRI studies suggested the frontal lobe is relatively less impacted in children with 22q11.2DS, more robust meta-analyses and recent endeavors nevertheless highlight it as a region of potential vulnerability when compared to TD controls [59,60,65,75]. Gothelf et al. [75] hypothesize that deficits in the frontal lobes may be related to the development of psychosis, other psychopathologies, and age-related declines in FSIQ. Neurodevelopmental and pathophysiological changes over time likely interact dynamically, confounding observations made at single cross-sectional timepoints. Despite the limitation of cross-sectional imaging, Rogdaki et al. [60] did find a significant moderate decrease in frontal lobe volume (g = −0.47) in their larger cross-sectional meta-analysis.

Anomalies in CT and SA are both observed in the frontal lobe in 22q11.2DS. Sun et al. [65], found significant focal CT thickening in the left caudal anterior cingulate cortex, as well as SA reductions in the anterior cingulate cortex and rostral middle frontal gyrus. Similarly, granular findings have emerged from other studies, for instance, focal decreases in CT in the cingulate cortex [62], CT increases in the middle frontal and orbitofrontal cortices [61], and SA reductions in the dorsolateral prefrontal cortex (DLPFC) and precentral gyrus [64]. The identification of these focal abnormalities in the frontal lobe may increase the specificity with which neuroanatomic variation can be tied to variability in executive functioning, emotional regulation, and abstract reasoning—all processes linked to the frontal lobe [76,77].

#### 3.3.2. Temporal Lobe

22q11.2DS is characterized by several structural anomalies in the temporal lobe, manifesting as both specific focal irregularities and broader trends in alterations of CV, SA, and CT. Bilateral grey matter and whole cortical volume of the temporal lobe have both been found to be significantly decreased (g = −0.84) in individuals with 22q11.2DS [60]. Localized volumetric reductions have also been found in the left inferior temporal region and temporal pole [62], parieto-temporal regions [64], as well as the inferior temporal cortex bilaterally and the fusiform gyrus [61]. The superior temporal gyrus (STG) is a region of particular interest given several studies have found either volumetric [78] or CT reductions [62,65,79]. Research has also identified increases in the left middle temporal gyrus [64] and right middle temporal region [61], supporting the finding of more generalized cortical thickening in the temporal lobe. SA reductions in the temporal lobe largely mirror increases in cortical thickness [61,62,65].

#### 3.3.3. Parietal Lobe

The parietal lobe, a region integral to sensory processing and spatial orientation, exhibits noteworthy structural irregularities in 22q11.2DS. Numerous studies underscore a pattern of decreased parietal lobe volume, with Rogdaki et al. [65] reporting a moderate to large effect (g = −0.73). Tan et al. [59], Schmitt et al. [62], and Jalbrzikowski et al. [61] also report volumetric reductions, particularly in the superior parietal cortex. Gudbrandsen et al. [64] found a somewhat more widespread impact within the parietal lobe. SA reductions have been noted in the superior parietal cortex [62,65], as well as postcentral and angular gyri [61,64]. Results for CT in the parietal lobe have been less consistent; both Schmitt et al. [62] and Jalbrzikowski et al. [61] observed CT increases in the inferior parietal lobe, whereas Bearden et al. (2007) reported reductions in the superior parietal cortices. Gudbrandsen’s [64] work further suggests an increase in CT in the pre- and postcentral gyrus and supramarginal gyrus. Structural deviations within the parietal lobe in individuals with 22q11.2DS may contribute to the observed deficits in spatial reasoning, mathematical abilities, and sensory integration characteristic of this condition [60,65].

#### 3.3.4. Occipital Lobe

The occipital lobe, long understood to be crucial for visual processing and spatial reasoning, is significantly impacted in 22q11.2DS. Studies converge on a pattern of reduced occipital volumes, with early research by Campbell et al. [56] noting decreased grey matter in the medial occipital cortex and overall volume reductions in the occipito-parietal cortex. These findings are supported by Schmitt et al. [62] who observed a significant volume reduction in the medial occipital lobe, as well as volumetric reductions in the cuneus by Jalbrzikowski et al. [61]. Both Tan et al. [59] and Gudbrandsen et al. [64] identified some occipital grey and white matter volume deficits. Schmitt et al. [62], Sun et al. [65], and Gudbrandsen et al. [64] identify particularly prominent SA reductions in occipital and occipitotemporal regions including lingual gyrus and in the medial occipital cortex. Similar to findings in the parietal lobe, occipital CT findings are somewhat less consistent between studies and do not always spatially correlate with SA deficits. Schmitt et al. [62] found an increase in CT in the cuneus and paracalcarine cortex, while Jalbrzikowski et al. [61] reported increased CT in the right lingual and left pericalcarine cortices. Bearden et al. [79] highlighted a significant CT reduction, particularly in the right parieto-occipital cortex, which was found to be 14% thinner than in TD controls, while additionally noting cortical thinning at the occipital poles. Gudbrandsen et al. [64] also found widespread increases in occipital CT, particularly in medial regions. These findings collectively underscore the occipital lobe’s relative vulnerability in 22q11.2DS, potentially related to the observed deficits in visually based cognitive processes associated with the deletion.

#### 3.3.5. Cerebellum

The cerebellum has historically been associated with a role in motor control functions, with individuals with cerebellar injury typically developing ipsilateral difficulties with motor coordination. However, over the last several decades it has been increasingly understood that the cerebellum plays important roles in the coordination of other brain functions including language, affect, cognition, and other higher-order functions [80,81]. Early MRI studies on 22q11.2DS did suggest statistically significant reductions in the vermis when measured in small samples via manual ROI tracings, with Eliez et al. [78] finding reductions in lobules VI and VII (superior posterior lobule) in 23 individuals with 22q11.2DS, and Bish et al. [82] describing significant reductions in the anterior lobule in 31 subjects. However, the cerebellar morphology in 22q11.2DS has subsequently been relatively understudied, in part owing to technical challenges in automating MRI image processing to the same degree as has been achieved for the cerebrum. With the advent of novel automated parcellation techniques, the cerebellum has reemerged as a structure of particular interest in understanding the neurocognitive profile of 22q11.2DS. Global cerebellar volume has been widely found to be significantly impacted in 22q11.2DS, with a meta-analysis by Rogdaki et al. [60] finding a large effect size (g = −1.25) in a sample of 147 subjects, and Schmitt et al. [83] finding a mean 17.3% reduction in total cerebellar volume in 79 individuals with 22q11.2DS compared to TD. Similar analyses have been carried out by Ge et al. [84], Tan et al. [59], and Campbell et al. [56], converging in their finding of global volumetric reductions in the cerebellum. Granular analyses of focal cerebellar deficits are more limited, though Schmitt et al. [83] identified volumetric reductions along an anterior-posterior gradient such that regions of the superior posterior cerebellum, Crus I and II, as well as lobules VIIB and VIIIA, were disproportionately affected after controlling for total brain volumes [83]. The most affected cerebellar regions are within the superior posterior lobe (SPL), the portion of the cerebellum most associated with higher cognitive function. With increasing appreciation for the cerebellum’s role in a variety of cognitive and behavioral functions implicated in 22q11.2DS, ongoing research will likely attempt to categorize the impact of granular volumetric deficits in tandem with the proven global trend.

### 3.4. Subcortical and Mesial Temporal Structures

The subcortical gray nuclei including the thalamus, putamen, and caudate demonstrate significant structural abnormalities in 22q11.2DS, with far-reaching associations with aspects of cognitive performance, behavioral functioning, and psychiatric comorbidity [85,86]. Several older studies have implicated the caudate in 22q11.2DS [56,87,88], a structure traditionally associated with extra-pyramidal motor function but (like the cerebellum) increasingly recognized for its involvement in higher-order functions including affect, learning, and memory [89,90]. Numerous studies have described ventricular abnormalities in 22q11.2DS, commonly reporting larger volumes relative to TD, with this finding potentially secondary to ex vacuo dilation from widespread cerebral volume loss [56,62,91,92]. In a large sample of 533 individuals with 22q11.2DS from the ENIGMA-22 consortium, Ching et al. [86] identified significant volume deficits in the putamen, and thalamus, as well as increased volumes in the caudate nucleus and nucleus accumbens. However, given the variability in reported patterns of volumetric abnormalities in some subcortical regions, likely colored by developmental and psychiatric factors [60,85], continued investigations of the 22q11.2 deletion’s impact on subcortical regions remains important.

Given its hypothesized association with schizophrenia [93,94], the hippocampus has received particular interest in 22q11.2DS. Eliez et al. [78] identified significant reductions in hippocampal volumes in children with 22q11.2DS using manual ROI tracings; decreased hippocampal size has subsequently been replicated in larger samples [39,60,95]. More recent higher resolution analyses in ENIGMA-22 have found that not only is the hippocampus the most significantly affected subcortical structure, but that there also are significant regional morphological abnormalities within the hippocampus with focal thinning along the medial-lateral axis and thickening along the dorsal-ventral axis [86]. Morphological patterns of thinning/thickening largely correspond to patterns within the cerebrum and may indicate aberrations in neuroanatomic connectivity will ultimately explain many findings in 22q11.2DS.

### 3.5. Developmental Trajectories and Comorbid Psychiatric Illness

Given that there is evidence that neurodevelopmental processes contribute to both 22q11.2DS and the onset of schizophrenia, it is somewhat disappointing that we currently only have limited information on the longitudinal neuroanatomic trajectories in 22q11.2DS. Despite the heterogeneity in structural findings among individuals with 22q11.2DS and the preponderance of cross-sectional data, we are slowly characterizing the dynamic changes in the functional anatomy of 22q11.2DS over the lifespan. For instance, we have identified accelerated cortical thinning in adolescents with 22q11.2DS relative to TD adolescents [64,96,97]. Gudbrandsen et al. [63] emphasize the need for contextualizing these findings based on developmental trends associated with individuals’ clinical phenotypes, with Biswas and Furniss [98] offering a review of the overlapping developmental periods in which particular psychiatric disorders commonly emerge in individuals with 22q11.2DS. While Sun et al. [65] noted considerable convergence in structural patterns in individuals with 22q11.2DS and psychosis compared to individuals with idiopathic schizophrenia, referencing many of the same trends categorized by Cheon et al. [85], the difficulty in disambiguating structural abnormalities specifically related to psychosis or those related to other comorbid disorders like autism spectrum disorder (ASD) is a persistent issue. In a comparison between idiopathic ASD and ASD in 22q11.2DS, Gudbrandsen et al. [63] note significant areas of divergence in both the neuroanatomical and behavioral phenotypes, demonstrating the potential interactions between comorbidities on patterns of neurodevelopmental change. Large prospective longitudinal analyses are imperative to delineate the patterns of morphological changes inherent to 22q11.2DS over the course of development and to identify those features’ associations with comorbid psychiatric conditions. Additionally, further replication studies on focal structural abnormalities linked to psychiatric disorders in 22q11.2DS are needed to address how these alterations may influence the overall neurocognitive profile, and how these regional differences may contribute to divergent behavioral profiles.

## 4. Neuroanatomic Underpinnings of Cognitive Dysfunction in 22q11.2DS

### 4.1. Structural Correlates of Overall Intellectual Functioning

Intellectual deficit, as measured by assessments of FSIQ, has emerged as the most common characteristic of the cognitive dysfunction seen in individuals with 22q11.2DS. As in non-deleted individuals, IQ in 22q11.2DS has been demonstrated to be a highly distributed characteristic in the number of discrete structures and structural patterns influencing the measure. Indicative of the broadest trends, Bearden et al. [99] found that global grey matter volume was positively associated with full-scale IQ in 22 individuals with 22q11.2DS. Furthermore, the authors note that temporal gray and white matter volumes were predictors of overall cognitive ability, a finding attributed primarily to their associations with verbal reasoning performance [99]. These results, while limited due to a small sample size, have been replicated in parallel studies in non-deleted populations that found medial temporal atrophy and global brain atrophy (as well as the prevalence of white matter hyperintensities) are associated with worse performance on assessments of overall cognition (e.g., Overdorp et al. [100]). All of these features, commonly found in Alzheimer’s, have also been documented to be highly prevalent in individuals with 22q11.2DS and point to global trends with a potential influence on overall cognitive functioning [91,100].

There is also limited evidence that polymicrogyria (PMG)—the presence of regional small gyri thought related to neuromigrational anomalies or prenatal insults –may be associated with cognition in 22q11.2DS. Although a relatively understudied endophenotype, clinical case reports have postulated its frequency to be higher in 22q11.2DS [91,101], and quantitative studies have shown regional anomalies in the gyrification index which may be related to polymicrogyria [67]. While limited case studies have noted heterogeneity in the presentation and severity of PMG in 22q11.2DS subjects, right-hemisphere perisylvian polymicrogyria, which has been associated with overall intellectual functioning deficits, speech production difficulties, and other motor deficits in clinical populations, was the most commonly identified subtype [102,103].

### 4.2. Structural Correlates of Executive Functioning and Attentional Processes

Individuals with 22q11.2DS demonstrate significant challenges in all central domains of executive functioning, including cognitive flexibility, inhibitory control, and working memory [30]. In addition, individuals with 22q11.2DS have a related, though likely etiologically distinct, deficit in sustaining and adaptively orienting attention [31]. Amid debate regarding the fundamental neuroanatomic organization of executive function, as well as a lack of a clear understanding of the structural underpinnings of attentional and executive capabilities in 22q11.2DS, research in this domain is critical [20]. However, the literature on this topic is currently sparse. A relatively rare study of executive functioning in 43 adolescents with 22q11.2DS found worse performance in real-world executive functioning was associated with increased CT in the right orbitofrontal cortex, working memory and sustained attention was associated with increased CT in both the right pars orbitalis of the inferior frontal gyrus (IFG) and the STG, and working memory and attentional control was associated with increased CT in the right precentral gyrus [104]. However, in the typically developing control group, the opposite trends between CT and behavioral measures were found. This discrepancy exemplifies the challenges in comparing brain-behavior associations between affected and control groups as the underlying neurobiology remains poorly understood and may meaningfully differ between populations. Within-group analyses somewhat circumvent this issue. A study by Dufour et al. [105] found that grey matter volume in the right cingulate gyrus in individuals with 22q11.2DS with low executive functioning was significantly lower than the in the individuals in the higher EF subgroup, which scored higher on the WISC III-Digit span subtest as well as the Stroop interference test assessing working memory and attention capacity as well as cognitive flexibility and inhibitory control, respectively.

### 4.3. Structural Correlates of Perceptual and Motor Skills

Neuroanatomical studies in 22q11.2DS have suggested distinct structural abnormalities in parietal and cerebellar regions are implicated in the visuospatial and motor skill deficits characteristic of the deletion. In a study of 71 adolescents with 22q11.2DS, Bostelmann et al. [106] identified deficits in overall visual memory performance in individuals with 22q11.2DS. Notably, a focal weakness in tasks relying on the ventral visual stream colloquially referred to as the “what” pathway, was identified in comparison to the dorsal “where” pathway, as evidenced by significantly poorer performance on visual-object memory and facial memory as compared to visuospatial memory. However, statistically significant associations between task performance and regions of the brain associated with the visual streams (e.g., inferior and ventral temporal cortices and the superior parietal cortex) were not observed [106]. The authors postulate that abnormal connectivity likely plays a central role in the observed cognitive deficits, perhaps why brain-behavior correlates were not identified using conventional MRI. Previous research has theorized posterior parietal dysfunction is also attributable to deficits in spatial attention and numerical enumeration [35], yet despite findings of focally reduced cortical volume in the parietal lobe [60] as well as localized regions of decreased posterior cortical thickness [79], the attribution remains to be thoroughly investigated.

Besides aberrant parietal functioning, the hippocampus has also been implicated in visuospatial memory, specifically in recalling the location and spatial relationships between entities [107]. However, hippocampal regions have similarly not been granularly analyzed to investigate specific visuospatial deficits in individuals with 22q11.2DS, despite replicated findings of volumetric decreases [39,60] and diminished thickness and surface area [86]. The fundamental neurological substrates of the established deficits in visuospatial memory in 22q11.2DS remain to be elucidated. Given visuospatial memory’s role in overall declines in perceptual abilities [34], characterizing implicated structures remains a critical endeavor. The degree of structural overlap between visuospatial deficits and motor abnormalities is perhaps most prominent in the cerebellum, specifically in its contribution to visuospatial memory deficits [83,108]. Boot et al. [109] document the heterogeneity in motor deficits experienced by individuals with 22q11.2DS as a result of cerebellar malformations that have been widely categorized in those with [60,83] and without [110] the co-occurring brain-wide changes associated with the deletion. Thus, while the interplay between neuroanatomical variations and perceptual deficits in 22q11.2DS needs to be further studied across a number of structures, the cerebellum’s pivotal role in both visuospatial and motor skills underscores the necessity for targeted research to delineate the underlying mechanisms of these associated impairments.

### 4.4. Structural Correlates of Language Processing Abilities

Verbal IQ and other language-related deficits are closely related to distributed structural abnormalities of the neuroanatomical profile of 22q11.2DS. Left cortical grey matter reductions, robustly documented in large meta-analyses [59,60], present as one of the broadest trends associated with decrements in verbal IQ [15]. Yet, findings by Shashi et al. [111] demonstrate the diffuse nature of verbal capacities, noting reduced grey matter volumes in the dorsolateral prefrontal cortex, cingulate, and cerebellum were each significantly associated with poorer verbal memory. Observed hippocampal volume deficits, in particular left hippocampal tail volume, have also been implicated in decrements in verbal learning, with authors additionally noting individuals with 22q11.2DS on the psychosis spectrum performed significantly worse on measures of verbal learning [39]. Other studies have also identified a relationship between psychosis and cortical thinning in key language regions, including the inferior frontal gyrus and superior temporal gyrus [65,79]. In a study of 72 adolescents, DeBoer et al. [112] further established a relationship between reduced hippocampal volume and reduced VIQ in individuals with 22q11.2DS. Additionally, Gudbrandsen et al. [63] found that within the 22q11.2DS subgroup with autism spectrum disorder, there were significant positive correlations between communication scores and the surface area of the left posterior cingulate cortex and the right temporo-parietal junction, reflective of these regions’ involvement in key language production and processing circuits and the communication deficits frequently observed in ASD [113]. Other imaging findings in these regions respectively demonstrate sharper declines in surface area in the posterior cingulate cortex across development [64] as well as the temporo-parietal junction’s role in attention shifting in social situations [114]. Together, these findings underscore the contributions of both regional and focal abnormalities in verbal processing and language deficits in 22q11.2DS, with the left hemisphere and numerous regions implicated in verbal memory and learning emerging as the most robustly categorized.

### 4.5. Structural Correlates of Socioemotional and Behavioral Dysfunction

The structural underpinnings of social, emotional, and behavioral deficits in 22q11.2DS remain to be comprehensively described; however, substantial analyses have been performed in individuals with 22q11.2DS using differential imaging paradigms [115,116], which, in conjunction with findings in phenotypically similar psychiatric populations [63,117], allow for some extrapolation as to the relevant structural trends. In a study of 42 individuals with 22q11.2DS, Glaser et al. [118] noted a differential pattern of volumetric abnormalities in the fusiform gyrus, implicated in facial identification and emotional processing, with an increase identified in anterior regions yet significant bilateral reductions in posterior regions. Similarly, volumetric deficits in the fusiform gyrus were identified by Jalbrzikowski et al. [61], with authors additionally noting the structure’s reduced surface area in comparison to controls. Related findings from an fMRI study in 22q11.2DS identified deficient activation patterns in a facial processing and emotional memory task aligned with findings of abnormal cortical surface area in the primary visual cortex, inferior temporal cortex, and dorsolateral prefrontal cortex (DLPFC) [119].

Functional imaging has also identified regions implicated in deficient social functioning and theory of mind-related capabilities in individuals with schizophrenia, somewhat mirroring those of normative populations, including the superior temporal gyrus, anterior cingulate cortex, and temporo-parietal junction, converging with structural abnormalities observed in 22q11.2DS [60,65,117,120]. Given the observed concordance of social functioning deficits between 22q11.2DS and schizophrenia [121], there is likely a high degree of parity in the underlying morphology driving these similarities. An alternative conception of social deficits, based on findings of deficient social cognition in 22q11.2DS being associated with emotional and behavioral dysregulation, implies a need for the consideration of additional behavioral features such as hyperactivity, disorganization, and internalizing symptoms in a search for the structural basis of prominent sociocognitive deficiencies [122].

Findings relating structural abnormalities to specific behavioral phenomena are limited; though in a study of 69 children with 22q11.2DS, reduced hippocampal volume and anterior hippocampal malformation were associated with anxiety in 22q11.2DS [123]. Potential downstream impacts on social functioning remain to be investigated. For many behavioral measures, there is strong phenotypic similarity in 22q11.2DS relative to non-deleted controls (e.g., schizophrenia, ASD), but disentangling which specific neuroanatomic variants are associated with specific behavioral deficits can be extraordinarily challenging, for example as previously mentioned for individuals with 22q11.2DS with and without ASD [63,64]. Cognitive-behavioral and neuroimaging studies have often identified robust and highly replicable patterns in 22q11.2DS, but attempts to link them have largely proven a disappointing exercise to date. It remains uncertain how commensurate the underlying structural mechanisms are between individuals with 22q11.2DS and a given psychiatric disorder compared to typically developing individuals with their corresponding idiopathic variants. Collectively, findings from different imaging modalities and populations underscore the extraordinarily complex relationships between neuroanatomic variation and the socioemotional and behavioral challenges observed in individuals with 22q11.2DS, pointing toward a multifaceted neurobiological etiology that necessitates further exploration.

## 5. Conclusions

The body of literature concerning 22q11.2 deletion syndrome has made considerable progress in characterizing both the distributed cognitive deficits and the neuroanatomical irregularities inherent to the syndrome. Individuals with 22q11.2DS often face widespread cognitive challenges, spanning holistic deficits in intellectual functioning, executive processes, attention, perceptual and motor skills, language processing abilities, and socioemotional functioning. These cognitive impairments are paralleled by a heterogeneous pattern of neuroanatomical alterations, encompassing global and localized changes in brain volume, cortical thickness, and surface area, alongside specific anomalies in cerebellar and subcortical structures. Yet, despite the advanced state of both largely independent pieces of literature, the current review highlights a pivotal gap in our comprehension of the precise structure–function relationships that underlie the distinct cognitive phenotypes observed in 22q11.2DS.

The present review underscores an urgent need for research that granularly delineates the intricate relationships between specific structural abnormalities and focal cognitive deficits characteristic of 22q11.2DS. Developing this understanding is critical for disentangling the complex interplay between multiple brain regions and cognitive dysfunction, and for identifying the relative impact of psychiatric comorbidity on the cognitive profile of 22q11.2DS. Future research should include both longitudinal and cross-sectional studies that explore structure–function associations with greater granularity and higher statistical power, employing both structural imaging alongside comprehensive cognitive assessments within large samples. By enhancing our understanding of the neural underpinnings of cognitive deficits in 22q11.2DS, we can not only improve the cognitive outcomes and overall well-being of individuals affected by the syndrome but also fully realize the utility of the deletion as a vital model for understanding many psychiatric disorders and for furthering our knowledge of the impacts of aberrant developmental processes.
